# Using a Bayesian hierarchical approach to study the association between non-pharmaceutical interventions and the spread of Covid-19 in Germany

**DOI:** 10.1038/s41598-023-45950-2

**Published:** 2023-11-02

**Authors:** Yeganeh Khazaei, Helmut Küchenhoff, Sabine Hoffmann, Diella Syliqi, Raphael Rehms

**Affiliations:** 1 Statistical Consulting Unit StaBLab, Department of Statistics, Ludwig-Maximilians-Universität, Munich, Germany; 2 Department of Statistics, Ludwig-Maximilians-Universität, Munich, Germany; 3 Institute of Medical Data Processing, Biometrics and Epidemiology (IBE), Faculty of Medicine, Ludwig-Maximilians-Universität, Munich, Germany

**Keywords:** Microbiology, Diseases, Health care, Medical research

## Abstract

Non-Pharmaceutical Interventions (NPIs) are community mitigation strategies, aimed at reducing the spread of illnesses like the coronavirus pandemic, without relying on pharmaceutical drug treatments. This study aims to evaluate the effectiveness of different NPIs across sixteen states of Germany, for a time period of 21 months of the pandemic. We used a Bayesian hierarchical approach that combines different sub-models and merges information from complementary sources, to estimate the true and unknown number of infections. In this framework, we used data on reported cases, hospitalizations, intensive care unit occupancy, and deaths to estimate the effect of NPIs. The list of NPIs includes: “contact restriction (up to 5 people)”, “strict contact restriction”, “curfew”, “events permitted up to 100 people”, “mask requirement in shopping malls”, “restaurant closure”, “restaurants permitted only with test”, “school closure” and “general behavioral changes”. We found a considerable reduction in the instantaneous reproduction number by “general behavioral changes”, “strict contact restriction”, “restaurants permitted only with test”, “contact restriction (up to 5 people)”, “restaurant closure” and “curfew”. No association with school closures could be found. This study suggests that some public health measures, including general behavioral changes, strict contact restrictions, and restaurants permitted only with tests are associated with containing the Covid-19 pandemic. Future research is needed to better understand the effectiveness of NPIs in the context of Covid-19 vaccination.

## Introduction

Severe acute respiratory syndrome coronavirus 2 (SARS-CoV-2) has quickly spread globally, with more than 38.4 million cumulative confirmed Covid-19 cases in Germany from the beginning of the pandemic by the end of May 2023, including a total of 174,170 deaths associated with SARS-CoV-2 infection^1^. Starting from March 2020, different bundles of non-pharmaceutical interventions (NPIs), at different times with varying stringency have been implemented to control the transmission of the virus. This was mostly done to protect the most vulnerable individuals from infection and to mitigate the surge of patients requiring hospitalization. By doing so, it aimed to protect the healthcare system from being overwhelmed by a sudden influx of cases. These NPIs included but were not limited to, containment measures such as domestic or international travel bans, individual protection measures like mask-wearing requirements, social distancing measures such as school closing and gathering bans, and health system measures like testing and contact tracing. All the NPIs are considered essential components of public health that people and communities can take to help slow the spread of illnesses^2–4^. However, the effectiveness of these policies remains a subject of debate, requiring further exploration to better understand the relationship between NPI intensity, duration, and their impact. It should be noted that, alongside these mandated policies, volunteer social behavioral changes were also observed during the pandemic. Thus the investigation of the impact of behavioral changes and concurrent NPIs is of immense importance.

In light of the severe social and economic costs^5^, affecting individuals’ behavior and mental health of these interventions^6^, it is crucial to quantify the effects of these measures. During the past two years, there were several attempts to identify the most influential measures across the world including Europe^4,7–14^. However, there is much discussion and controversy around the matter. In 2022, Rehms et al. proposed a Bayesian hierarchical approach, as a common framework, that integrates disease incidence, hospital occupancy, and mortality, as complementary sources of information to get a reliable estimate of the unknown number of infections. The model takes into account that published data suffers from time-varying under-reporting and reporting delays. Moreover, effects on the disease dynamics over time are incorporated: The effect of vaccinations starting on the 8th of December, 2020, and the rise of new variants of concern, which accelerated the spread of the virus and increased its lethality. By explicitly modeling these characteristics, it is possible to look at a larger time horizon and therefore utilize more data, making the results more robust. Hence, one is not forced to look at small periods where constant disease dynamics can be assumed to justify simplified models^15^.

Germany has implemented different containment and mitigation strategies starting in March 2020. In this paper, we apply the proposed Bayesian hierarchical approach for all sixteen states of Germany, for a time period of 21 months, and evaluate the effectiveness of different NPIs.

## Materials and methods

### Data sources and preprocessing

#### Selection of NPIs

This study includes various NPI time series obtained from the Corona data platform. A team of researchers from different institutes, including the Corona data platform, Infas, Infas 360, and the University of Bonn, funded by the Federal Ministry of Economics and Climate Protection of the Federal Republic of Germany collected regional data on an ongoing basis of all measures and epidemiological-medical as well as socio-economic indicators of all cities and districts^16^. From 23 categories and 1152 subcategories^17^, 8 main measures, across 16 different states are selected: “contact restriction (up to 5 people)”, “strict contact restriction”, “curfew”, “events permitted up to 100 people”, “mask requirement in shopping malls”, “restaurant closure”, “restaurants permitted only with test”, “school closure” and one additional NPI labeled as “general behavioral changes”. Rehms et al. proposed this NPI to account for changes in people’s behavior during the pandemic. This NPI is active all the time starting with the activation of the first NPI^15^. It is important to account for such an effect as it makes the other NPIs more comparable (e.g. closing schools or restrictions on gatherings will not have a comparable effect if social distancing in case of respiratory symptoms is practiced). It can therefore be seen as a residual for untracked or latent NPIs which are not directly implemented by the government and are implicitly active through behavioral changes in the population.

The reasons for which we selected this set of NPIs are threefold: they either reflect the characteristics of a specific time span of the pandemic (curfew, events permitted up to 100 people, restaurant closure, restaurants permitted only with test, school closure), they enable us to compare two specific measures with different strictness level (contact restriction (up to 5 people), strict contact restriction), or they evaluate the effectiveness of more long-lasting measures such as mask requirement in shopping malls. Note that not all of the interventions were implemented in all the states. We define the interventions as presented in Table 1.

**Table 1:** Description of the defined non-pharmaceutical interventions (NPI).

Name of NPI	Definition
Contact restriction (up to 5 people)	Max. 5 people, except a household and close family members (private and public settings merged together)
Strict contact restriction	Only persons of a household and close family members (private and public settings merged together)
Curfew	Exit restriction; leaving the apartment only for a valid reason
Events permitted up to 100 people	Indoor public events, up to 100 people
General behavioral changes	This NPI captures many behavioral adaptations people took during the pandemic and is active from the first time an NPI was implemented in a state and remains active until the end of the observation period. The list includes but is not limited to wearing masks, increased engagement in positive/negative health behaviors, working from home, less physical contact, and generally higher vigilance in terms of one’s personal health
Mask requirement	In shopping malls and sales outlets
Restaurant closure	Catering establishments of any kind are prohibited. The sale and delivery of takeaway meals are exceptions
Restaurants permitted only with tests	Test-related access restrictions
School closure	Primary/secondary schools and partial/complete school closures merged together (selection of 1 final class and grade, or selection of 2 final classes and grades, or emergency selection of 3 classes and grades for children of certain parent groups, or selection of 4 teaching sessions of only certain subjects). All school holidays for each state were added manually

#### Reported cases, hospitalizations, and deaths

We use the following official data sets: data on reported cases, hospitalizations, and deaths in Germany are published daily by the RKI on a state level^18^. The RKI is a German federal government agency and scientific institute responsible for health reporting, disease control, and prevention. As the national register for Covid-19, it preserves all identified disease cases reported by the local health authorities. In our analysis, we use daily reported cases, the number of new patients admitted to hospitals due to Covid during the past 7 days, and a daily number of deaths due to Covid-19, on a daily basis for each state.

#### Intensive care unit occupancy

Data on the daily occupancy of Intensive Care Unit (ICU) beds in Germany is made publicly available by the German Interdisciplinary Association for ICU Medicine and Emergency Medicine^19^.

### The hierarchical model

In this section, we provide a short description of the used model. A more technical description is given in the Supplementary Material Section B. To estimate the effect of the NPIs, we use a Bayesian hierarchical approach proposed by Rehms et al.^15^. Hierarchical models provide a flexible framework to describe complex phenomena through the combination of different submodels. Hereby, each submodel handles another small part of a big problem, making the intractable tractable. To apply the model to German data, we modify the proposed model, making it more flexible and tailored to the data. In the following, we give a short description of the model and its modification. For more insights on the methodological aspects, we refer to the original work of Rehms et al.^15^.

The model can be divided in two constituent parts: The first one infers the number of infections for each time point and region from given data. The second one infers, given these infections, the effect of the NPIs. The actual true number of infections can not be observed directly, as official numbers suffer from incomplete and delayed reporting, variations in testing strategies, and more. To infer these actual infections over time, the proposed model uses four different time series as complementary sources of information: reported number of deaths, cases, and the occupancy of hospital beds and ICUs. Each of the series provides individual information on the disease dynamics. For example, one could use the reported deaths to ’calculate back’ the number of infected individuals. We do this with all four series linking each of them through individual submodels. As we use a probabilistic Bayesian approach, the uncertainty about each of the linked models is preserved in the inferred number of infections. Given these inferred infections for every day in every region, it is possible to estimate the effect of the NPIs using a renewal equation (see e.g.^20^). The renewal equation formulates the disease dynamics as a function of the reproduction number and the past infections. It can be seen as a flexible version of classical compartment models like the SIR model^21^. The reproduction number in this renewal equation is formulated as a function of a basic reproduction number and the effect of the NPIs. This quantities are then estimated within this framework.

To derive the unobserved infections, the model takes into account that data are reported with delays and suffer from seasonal and structural under-and over-reporting due to weekends and varying testing policies. Moreover, the model does not rely on constant dynamics over time: With the surge of new variants of concern, the contagion process and the infection fatality rate (IFR) change. Both quantities are also affected by the vaccination coverage of the population. By including all these factors, it is possible to use a much larger time horizon, resulting in more usable information to reliably infer the effect of NPIs. It is therefore not necessary to focus on short time periods during which disease dynamics and the degree of underreporting can be assumed to have remained constant. To get estimates of the NPIs, the model is designed in a hierarchical way: The effect of each NPI is estimated for each location (here, the federal states of Germany) separately while sharing a common mean and standard deviation. Therefore, the parameters can borrow information from each other while allowing for an individual effect for each location. The hierarchical formulation gives robust and reliable estimates of the NPIs’. Besides the effect of the NPIs, the effect of the seasons as a proxy for behavioral changes of the population with respect to drifts in weather and temperature are estimated as well. To fit the model of Rehms et al.^15^ to the German data, we modify it in two ways. Firstly, as Germany does not provide data about hospital occupancy, we use hospital admissions (for ICU, occupancy data were available). The time-shifting distribution to be used is simplified, as it is not necessary to model the time of occupancy. Secondly, we introduce a new parameter that allows for a change in the fraction of hospitalizations and ICU occupancy on the 1st of July 2020. This gives the model more flexibility to estimate the relationship between hospital data and unobserved infections. As there was only limited experience with COVID-19 in the first wave, the hospitalization pattern may have changed afterwards.

### Data preparation

Official data for the first months of the pandemic were not available on the state level. We impute these data points as follows: We considered the sum of the first two weeks of the available data on reported deaths (as it is the most reliable source of information) and calculated the relative proportion of each state compared to all reported deaths in Germany. As aggregated data on the country level were available from the start of the pandemic, we use these estimated proportions to split this aggregated data into the theoretical number of reported deaths and cases for each German state (rounded to integers). This procedure implies that at the beginning of the disease, the infection dynamics are the same in each federal state. Following Rehms et al.^15^, we define the start of the observation period in each state as 30 days before the number of reported cumulative deaths reaches or exceeds a count of ten. The first considered dates range from the 18th of February 2020 for Bavaria to the 8th of March 2020 for Mecklenburg-Western Pomerania. The median of the considered days is 615. The observation period ends on 31 October 2021. The merging process of “Berlin & Brandenburg”, “Bremen & Niedersachsen” and “Hamburg & Schleswig-Holstein” is described in Supplemental A.1 Section.

### Conducting Bayesian inference

The proposed model requires the definition of many different parameters (fixed and variable). To get sensible specifications, we use the same definition as Rehms et al.^15^. Fixed quantities are taken exactly as the specification, as for Germany, and deployed for all states of Germany. This assumes, for instance, that the infection fatality rate for two persons who are doubly vaccinated and getting infected with the alpha variant of the virus at the same time is the same, regardless of their location. Prior distributions are also set in the same way as in Rehms et al.^15^.

Inference of the proposed model is done via Markov Chain Monte Carlo as described in detail in Rehms et al.^15^ using a customized Metropolis-Hastings update scheme tailored for the model^22,23^. We run eight chains with 50,000 iterations. Beforehand, each chain used 20,000 samples as burn-in. We only keep each 100th value of each sampled Markov chain to reduce autocorrelation, resulting in a final sample of 4000 data points from the posterior distribution. The presented results are the derived empirical quantities from this model.

## Results

We fit the model to our dataset, which included the list of NPIs (as described above), coded as dummy variables with the earliest start at 2020-02-18 until 2021-10-31 for 13 federal states. Each row was complemented by the data on the number of reported cases, hospitalizations, ICU occupancy, deaths, vaccination coverage, seasons, and variants of concern. Using our model, we were able to calculate the effectiveness of each NPI in reducing the instantaneous reproduction number as a percentage value for each measure.

**Figure 1:**
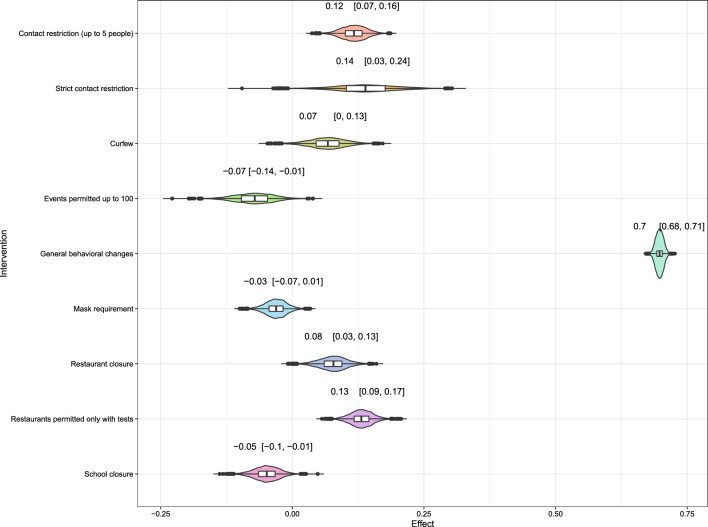
Estimated effects of the defined NPIs. The x-axis gives the relative reduction in % (obtained with the transformation $1-\exp (-\alpha _k)$ from the original estimated values). The y-axis indicates the defined NPI. The colored area shows the distribution of the estimate. The number above each row shows the estimated mean effect along with a 95%-credible interval. As the numbers indicate a relative reduction, a negative value can be interpreted as a *relative increase*.

Figure 1 shows the estimated effects of the NPIs as a reduction or an increase in % of the instantaneous reproduction number with a 95% credible interval. The effects are presented as the mean effects over all states. The largest effect is given by general behavioral changes, which reduces the reproduction number by 70% (CI: (68%, 71%)). This effect captures non-observable effects which are not encoded by other NPIs. The effect in the reproduction number was followed by a significant reduction with strict contact restriction by 14% (CI: (3%, 24%)), restaurants permitted only with tests by 13% (CI: (9%, 17%)), contact restriction (up to 5 people) by 12% (CI: (7%, 16%)) and restaurant closure by 8% (CI: (3%, 13%)). Curfew showed a marginal effect by reducing the reproduction number by 7% (CI: (0%, 13%)), while events permitted up to 100 people showed an increase in the reproduction number by 7% (CI: ($-$14%, $-$1%)). This increase was observed in school closures by 5% (CI: ($-$10%, $-$1%)) and mask requirement in shopping malls and sales outlets by 3% (CI: ($-$7%, 1%)) as well, albeit not statistically significant. Furthermore, we estimate the effect of the season as a nuisance parameter. One can interpret its result as a relative change to the summer months, which serves as a reference category. We observed a significant negative effect for autumn (an increase in the reproduction number by 28% (CI: ($-$33%, $-$23%)) and for winter (by 7% (CI: ($-$13%, $-$1%)). Spring reduces the reproduction number significantly by 11% (CI: (7%, 14%)).

**Figure 2:**
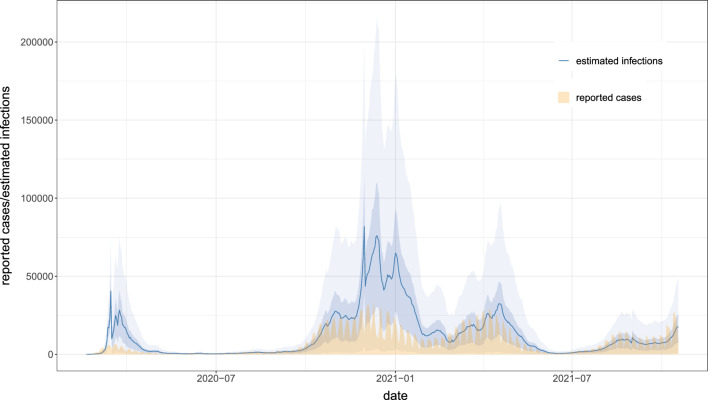
Estimated infections with 75%- and 95%- credible intervals in blue. In yellow we provide the reported cases for a better comparison. We shifted the reported cases by 13 days to the left to get a better comparison between the two curves. This shift reflects the mean time until an infected person is getting symptoms (roughly 6 days) and being reported as an actual case (roughly 7 days) afterward.

To test the robustness of the results, we also made three sensitivity analyses by varying crucial parts of the model that could influence the results. However, we found no substantial difference in the estimated effects. Besides the results relating to the NPIs, the model also estimates the unknown number of actual infections in all federal states. We show an aggregated version of these estimations in Fig. 2, i.e. the sum of the estimated infections over all federal states with a 75%- and 95%- credible interval in blue. For comparison, we also provide the reported cases (also aggregated to country level), but shifted by six plus seven days to account for the incubation time and the time until a case is actually reported. The estimation of the infections implies higher under-reporting in phases of large growth rates, in particular in the first and second wave of the pandemic. This effect seems to vanish around July 2021.

## Discussion

Since the beginning of the Covid-19 pandemic, many recommendations have been made for citizens and several social measures have been implemented. Besides vaccination, NPIs play a unique role in preventing the pathogen from being transmitted. Previously, the effectiveness of NPIs across different countries was extensively reviewed. Here, we used a data-driven approach to estimate the effects of nine NPIs, from March 2020 to October 2021, in Germany. General behavioral changes were associated with the largest reduction in the effective reproduction number, followed by measures including strict contact restriction, restaurants permitted only with a test, contact restriction (up to 5 people), restaurant closure, and curfew. The current work showed that some NPIs were associated with a clear reduction in the instantaneous reproduction number, which was consistent with the increasing evidence indicating that NPIs are efficient in alleviating and controlling Covid-19 outbreaks. However, some NPIs showed mixed results compared with the existing literature.

We believe that by using the proposed Bayesian hierarchical approach, we can integrate different data sources and this has several benefits such as increasing the amount of data available for estimating NPI effects and reducing biases in the reporting of cases and deaths. Since we are not attempting to deduce the total number of Covid-19 infections, our results are more robust to violations concerning the assumptions about specific infection fatality rates (IFR). Lastly, we allow the effects of all NPIs to vary across states, acknowledging differences in NPI implementation and adherence.

There are some factors that limited our analysis of the estimation of NPI effects. First, defining NPIs proved to be a complex task. According to the Corona data platform, an NPI variable was considered active if the measure was in place at the federal-state level. This information, while exceptionally detailed and organized, was also dependent on the 7-day incidence rate, potentially resulting in a weekly pattern for NPI activation, as outlined in the Supplementary Material Section A. Therefore, we implemented a rigorous decision-making strategy to extract the NPI data. Second, many measures were introduced simultaneously (e.g., the introduction of mask requirements and the prohibition of mass events). Talic et al. noted a similar challenge, where over half of the 72 studies they reviewed couldn’t be included in their meta-analysis because they evaluated “packages” of measures in the form of NPI combinations, making it impossible to assess the effects of each NPI individually^24^. On the other hand, the imposition and relaxation of control measures varied across federal states. For instance, contact restrictions limiting gatherings to a maximum of 5 people in private settings were enforced in Baden-Württemberg in mid-March 2020, while the same restriction was applied in Bavaria in mid-May 2020. Similarly, we anticipated substantial variation in school closure measures since we accounted for school holidays, which are not uniform nationwide across Germany, meaning it is not the same in different states. Consequently, decisions on closing schools were sometimes made in accordance with school holidays; leading to instances of no implementation of school closures provided a holiday in place or sometimes merely extensions of existing holidays. Given the complexities described above, through the hierarchical formulation of the model, it is possible to identify the effect of an NPI, as long as there is some degree of heterogeneity among different locations. A third limitation in our analysis pertains to potential interdependencies among infection dynamics in different states. Unfortunately, our model cannot account for these potential dependencies.

The key strength of this study is twofold. First, we were able to use high-quality and comprehensive daily data on reported cases, hospitalizations, deaths, and occupancy of ICU beds provided by RKI and DIVI through their respective dashboards. Furthermore, a critical asset to our analysis is the Corona data platform, which consistently delivers detailed information on the implementation of NPIs at the state level on a daily basis. This granular dataset empowers us to define NPIs with precision, a fundamental requirement for our investigation. It is worth noting that the data on the platform is methodically based on the Oxford Stringency Index^25^, a recognized metric for assessing governmental responses to the pandemic. Since March 2020, the platform has systematically collected official publications pertaining to Covid-19 protective measures and diligently categorized their content into various upper and subcategories. While our study shares a hierarchical data structure with the Oxford Stringency Index, it distinguishes itself through the depth and the content of the respective coding^17^. The second strength is the use of a sophisticated model that comprises a wide variety of aspects, including but not limited to the use of the information from four different daily time series (reported cases, hospitalizations, deaths, and ICU occupancy) to infer reliable disease dynamics. While the model accounts for uncertainties in the information (e.g. under-reporting), it also considers effects like vaccinations and the emergence of new variants of concern making it possible to use information over a relatively long period of time giving more informed estimates. Moreover, we do not need to smooth the observed time series, since we account for variations in daily reporting patterns in reported cases and deaths.

The roles of general behavioral changes or measures and their public adoption during a pandemic have been evaluated before^26–28^. The term general behavioral changes encompasses any actions that contribute to reducing the transmission of Covid-19 and, as a result, aid in containing the pandemic. Hence, this NPI subsumes a large variety of not directly defined NPIs which are more latent and are very difficult to define on an aggregated level. It can include various practices, such as practicing good hygiene by washing or disinfecting hands, following proper cough and sneeze etiquette, and regularly cleaning surfaces. Additionally, it involves engaging in voluntary physical distancing measures, such as staying at home, limiting close contact, and avoiding crowded places. Wearing masks or gloves, staying home when experiencing respiratory symptoms, utilizing testing services, refraining from non-essential travel, and utilizing contact-tracing applications are among other measures included within this term. The definition of general behavioral changes can therefore be quite vague depending on the considered context of a study as it may include some of the mentioned aspects or not. In this work, the NPI general behavioral changes serve as a controlling variable, making other NPIs more comparable to each other by capturing latent NPIs that are not directly defined or implemented. It is therefore important, to interpret the result for this variable with caution. More often than not, general behavioral changes or measures are under-evaluated and their consideration in the epidemiological models is limited^26^. In an SEIR model suggested by Khairulbahri, they reported a reduction in infected cases of about 22%, by studying behavioral measures effect, which was consistent with the findings of our study^27^. A reduction in the incidence of Covid-19 associated with physical distancing (75%, CI: (59%, 95%)) was reported in a meta-analysis by Talic et al., which is in line with the results of our study^24^. In addition, Brauner et al. showed limiting gatherings to fewer than 10 people had a large effect size for reducing transmission at the advent of the pandemic (42%, CI: (17%, 60%))^29^. We found similar substantial reductions in the reproduction number for restaurants permitted only with tests and restaurant closure. Ledebur et al.^30^ reported restrictions in gastronomy reduced transmissions by about 17%. However we should keep in mind that their analysis focused on less disruptive measures that did not consist of full closures, but rather of restrictions such as mandatory registration of visitors, limits for the opening hours, or the number of people seated at a table. However, on the same note, the effectiveness of fully closing gastronomy has been repeatedly established in the literature^31–33^.

We observed marginal effects for cancellation of events beyond 100 people and curfew in our results, which should be interpreted with caution. Previously, in a study on the effectiveness of a nighttime curfew in Hamburg, Germany, the researchers concluded that the curfew was substantially reducing the number of Covid-19 cases^34^. Several other studies found that nighttime curfews reduce mobility, hence they result in fewer Covid-19 infections^35^. It should be considered that we included curfew as an exit restriction; leaving the apartment only for a valid reason, which is considered a harsher intervention than a nighttime curfew. In detail, curfews and cancellation of events beyond 100 people were implemented only over short periods across many states, and mostly, in co-treatments with other NPIs implemented at the same time. This endogeneity of the policies can hinder the estimation of the true effectiveness of NPIs. On the other hand, it has been discussed that strict exit restrictions that limit the lives of citizens might backfire and increase Covid-19 infections^36^. However, there is little and rather mixed evidence on the effectiveness of curfews with varying strictness to contain the Covid-19 pandemic.

Our study showed no evidence for the effectiveness of school closure. Previously, Talic et al. were not able to make a consensus for school closure, due to the high heterogeneity between studies. They qualitatively reported that school closure could be highly effective if implemented early, with low incidence rates of Covid-19^24^. This is in accordance with Fritz et al who emphasized that the effectiveness of school closure in the case of Covid-19 is inconclusive and high caution should be maintained when interpreting the results, specifically due to many socio-economic and psychological implications to it^37^. Similarly, the same result was reported by Rehms et al. They estimated the smallest effect for school closure with a credibility interval that included zero^15^. Moreover, Isphording et al. showed the number of Covid-19 infections did not increase with school re-openings in the summer of 2020^38^. All in all, with the simultaneous implementation of different public health measures, the results should not be overstated^24,39,40^.

Thoroughly examining the effectiveness of interventions presents relevant obstacles in terms of methodology. While simulation studies can investigate different situations, they rely on strong assumptions that may not be easily verifiable and bring a low level of evidence^41,42^. As an alternative approach with potential, we used cross-state modeling that is data-driven and compares the timing of state-wide interventions with the subsequent cases, hospitalization, ICU, or death counts. In the previous works, there was a fairly large variation among the inclusion of different sets of NPIs and methodologies in use. They reported varying results on the effectiveness of public health measures in reducing different outcomes such as incidence, transmission, or mortality. Hence, the comparison between these studies can be impeded by this variation. Talic et al. mainly used observational studies from different countries in their meta-analysis. They further explained the concern hovering around the ability of the mathematical models and their assumptions, to predict the course of virus transmission or the effectiveness of interventions was the main reason they excluded such studies in their meta-analysis^24,43^. Additionally, sophisticated and flexible methods, like Bayesian longitudinal models, were used by some researchers^9,29,44^. For instance, Hunter et al. used Bayesian generalized additive mixed models to adjust for spatial dependency in Covid-19 between nation states, as well as multilevel mixed-effects negative binomial regression model with cases or deaths on a specific day as the outcome variable^45^. Some studies used linear regression, simple correlation coefficients^41^, or mixed effects linear regression^42^.

Moreover, different outcomes of interest have been reported as well, including the number of confirmed cases, mortality or death rate, or confirmed deaths. Bo et al. evaluated the effectiveness of four types of NPIs on the transmission of Covid-19 by generalised linear mixed model, with city/country-level random intercept in the model to control for clustering effects within the same city/country^46^. At the same time, focusing on short time periods during the pandemic or using either the number of reported cases, intensive care occupancy, new hospital admissions, or deaths, as a single indicator of disease transmission, would give an incomplete picture of the pandemic. All things considered, the settings, methodologies, and results of these studies were inconsistent and the interpretation and application of their findings and methods should be done cautiously. Banholzer et al. pointed out that such huge variation in a plethora of published studies can result in the robustness of the results in different settings, as much as it can hinder conclusive evidence on the effectiveness of NPIs^44^.

In conclusion, the current work contributes to the body of evidence on the effectiveness of individual NPI. As previously mentioned, serious deficiencies in the available empirical data are observable. Although our work focused on a data-driven approach to estimate the effects of NPIs, our estimates should not be taken as the final word on NPI effectiveness. Further high-quality original studies with reliable effect estimates are necessary in order to avoid unrealistic expectations or overestimation of the effectiveness of the NPIs.

### Supplementary Information

Supplementary Information.

## Data Availability

Supplementary materials for this paper are available from the publisher’s webpage including additional information on data preprocessing, technical description of the model, and results of sensitivity analyses. The code to run the model is available in the following repository: https://github.com/RaphaelRe/COVID_NPIs_Germany.
