# Supplementary material for: Using a Bayesian hierarchical approach to study the association between non-pharmaceutical interventions and the spread of Covid-19 in Germany
Source: Sci Rep. 2023 Nov 2;13:18900. doi: 10.1038/s41598-023-45950-2 (PMC10622568; doi:10.1038/s41598-023-45950-2)
Supplement: Supplementary file 1 — Supplementary Information. [file 41598_2023_45950_MOESM1_ESM.pdf]

Supplementary Material

A Data sources and preprocessing

A.1 Underlying Covid-19 data sources

In this section, we provide graphs of the underlying COVID-19 data sources: reported cases (Figure S1), deaths (Figure S2) hospitalizations (Figure S3) and intensive care unit occupancy (Figure S4) by state.

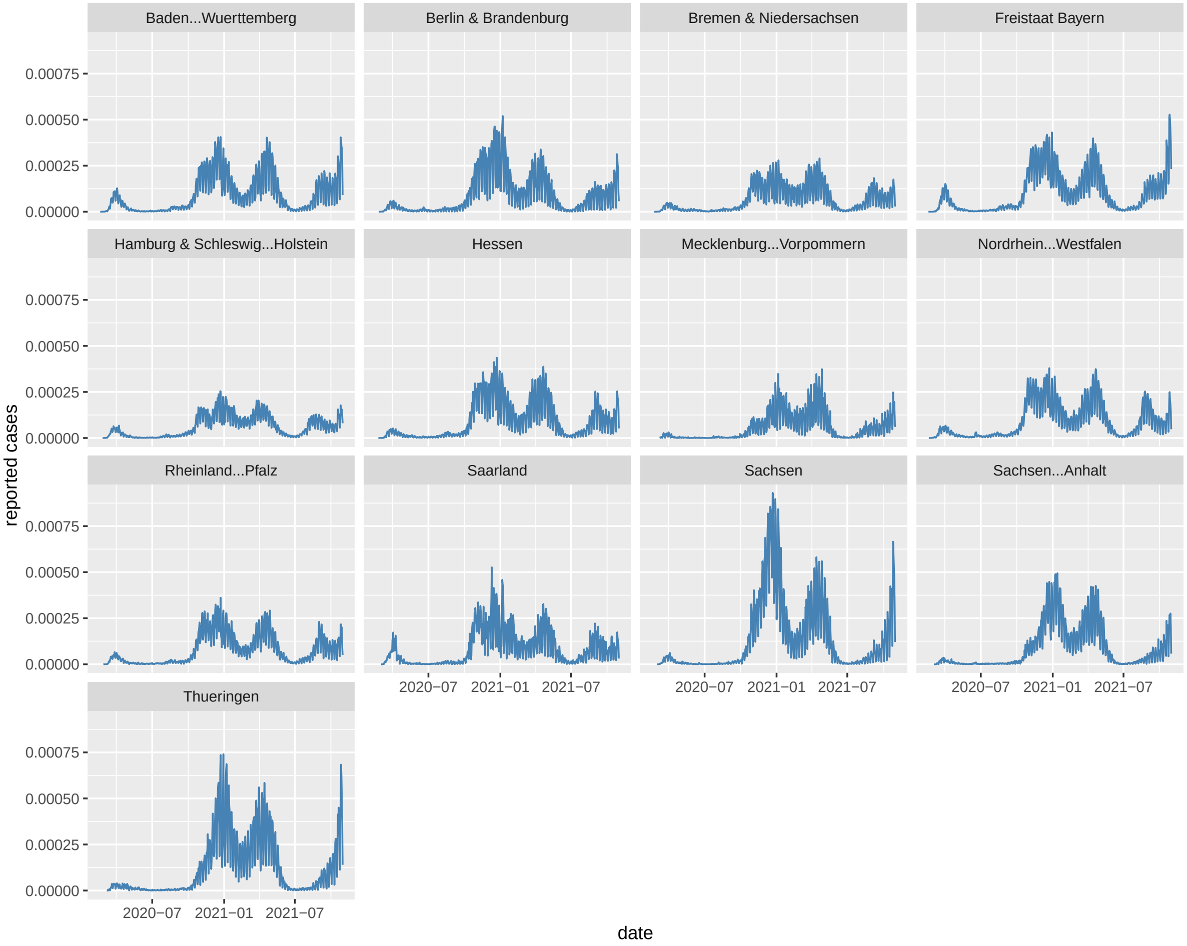

Figure S1: Number of Covid-19 reported cases as a proportion of the population in each state

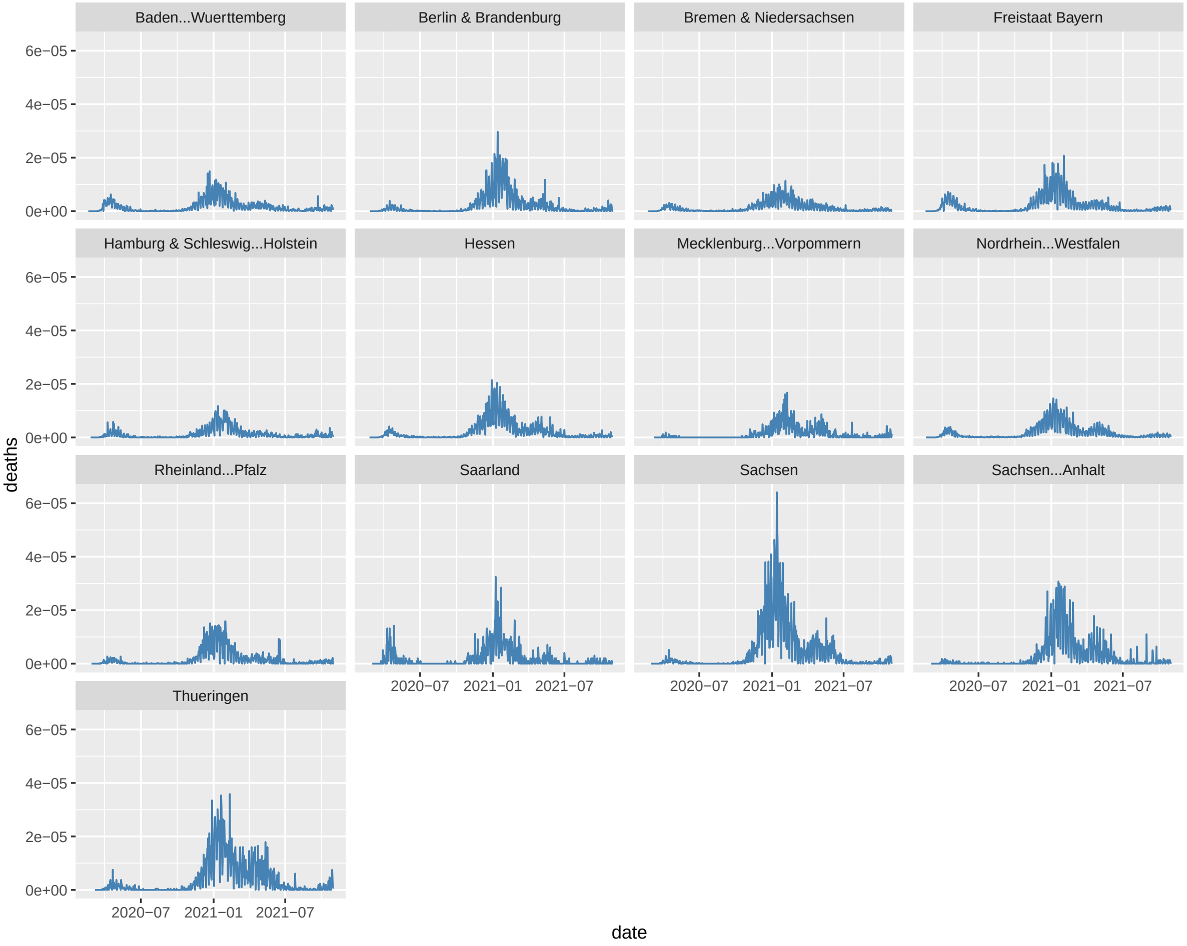

Figure S2: Number of deaths due to Covid-19 as proportion of the population in each state

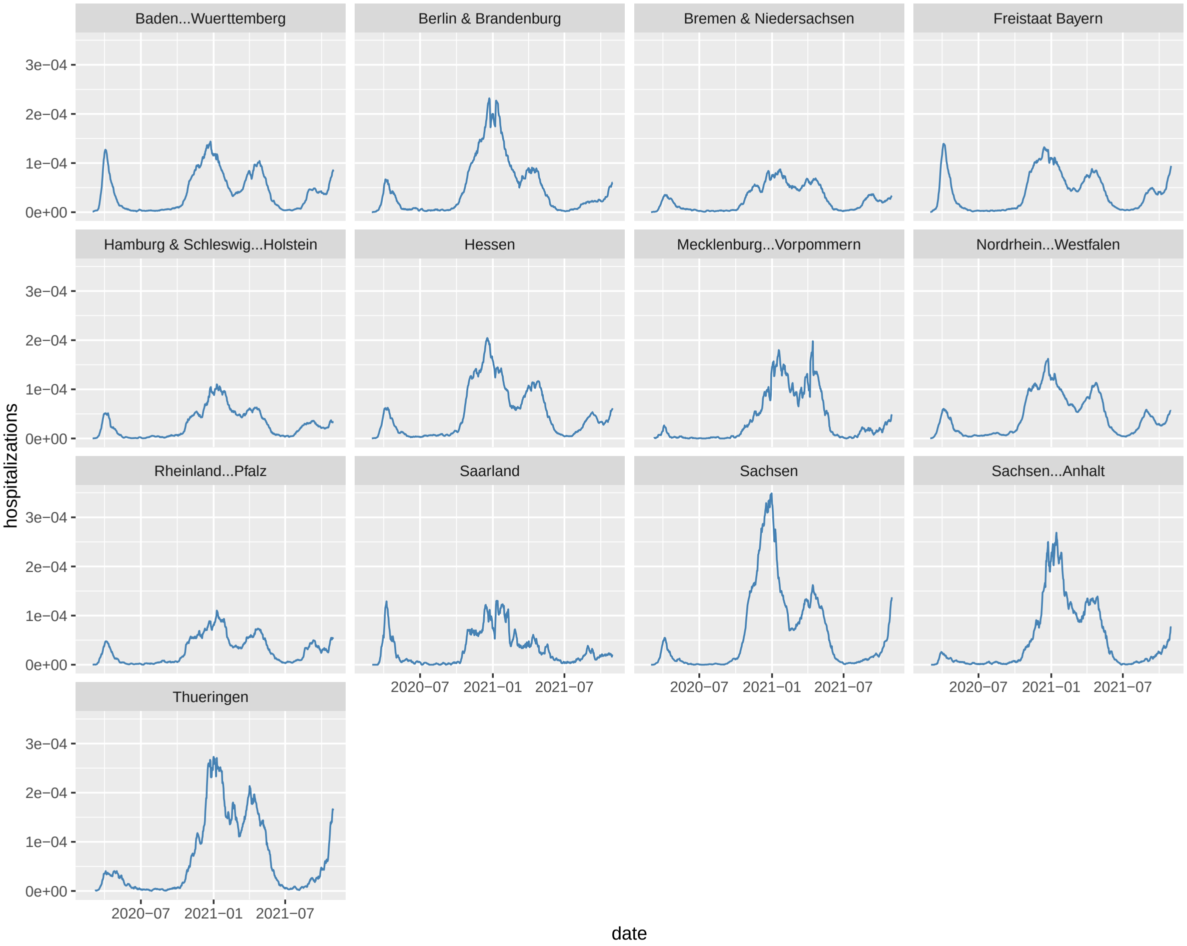

Figure S3: Number of new patients admitted to hospitals due to Covid-19 as proportion of the population in each state

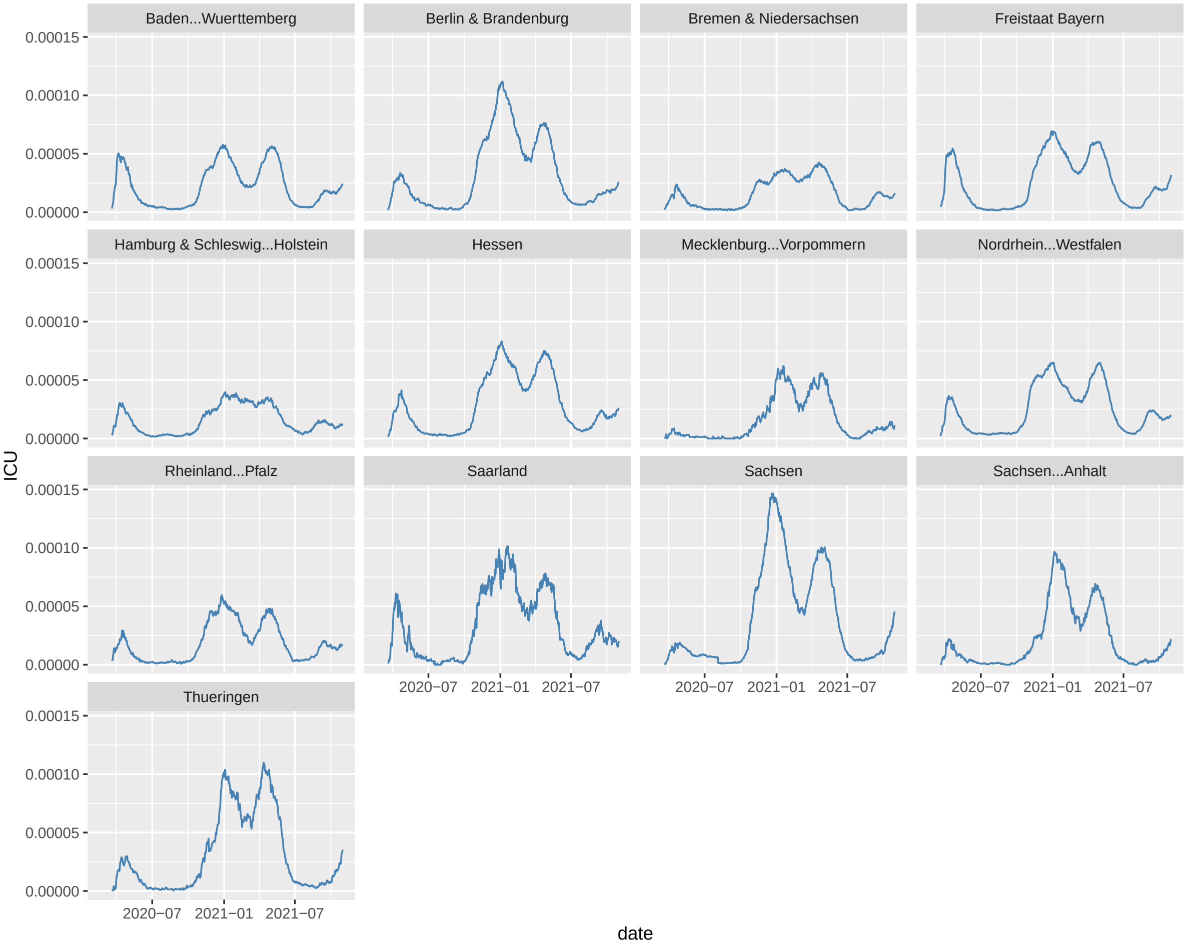

Figure S4: Number of occupancy of Intensive Care Unit beds due to Covid-19 as proportion of the population in each state

A.2 NPI definition

The data provided by the Corona data platform is methodically based on the Oxford Stringency Index [25]. Official publications on corona protection measures have been collected there since March 2020 and their content was coded into various upper and subcategories. A similarity to the Oxford Stringency Index lies in the hierarchical data structure. There are main categories that represent the different areas that are affected by the various regulations, such as schools, retail or cultural institutions. The subcategories describe the specific measures, such as closures or partially restricted openings with certain conditions. However, the difference lies in the content of the respective codes and the depth of the coding. For example, while the Oxford Stringency Index covers all school types and universities in the "School Closings" area, the Index we used divides schools into primary schools and secondary schools. There are also additional categories that divide the measures into public or private areas. Another difference is the regional depth of the researched ordinances.

Corona data platform provided the data on each NPI in a separate Excel file per request. Each data file consisted of a federal state name, a date starting from the 1st of March 2020, and a list of subcategories pertaining to each NPI. The subcategories include:

- Independent of new infections;
- From 0 new infections, per 100K;
- From 10 new infections, per 100K;
- From 35 new infections, per 100K;
- From 50 new infections, per 100K;
- From 100 new infections, per 100K.

Each of these subcategories can be indicated by "0: Measure not mentioned, 1: measure available, 2: Imputation of measures, and -99: Measure is no longer used". We decided not to use the imputed measures and exclude -99 as NAs.

The sum of each row was calculated indicating that a specific NPI was implemented if any of the subcategories were indexed as 1. "Berlin & Brandenburg", "Bremen & Niedersachsen" and "Hamburg & Schleswig-Holstein" were merged as well, i.e. if NPI x on date x in Berlin was 0 and it was 1 for Brandenburg, the mean was 0.5, which was set to 1 for "Berlin & Brandenburg".

The decision of including the subcategories and/or the imputed data came after comparing the results of each scenario (inclusion of imputations and subcategories, inclusion of imputations without subcategories, inclusion of subcategories without imputations, exclusion of both) was matching the reality, i.e. if our preprocessing steps resulted in the implementation of NPI x on date x, conditional on 35 new infections per 100K, whether it really happened. This could be tested by comparing our results with the incidence data published by RKI.

We opted for the dataset including NPIs across 13 states as mandates (not recommendations), when imputation was set to 0 and the subcategories were included, as shown in S8. For school closure, we incorporated school holidays into our NPI definition. Unlike school holidays, weekends are not incorporated into this NPI, since we believe contact patterns can be expected to change a lot during holidays and school closures but not during weekends.

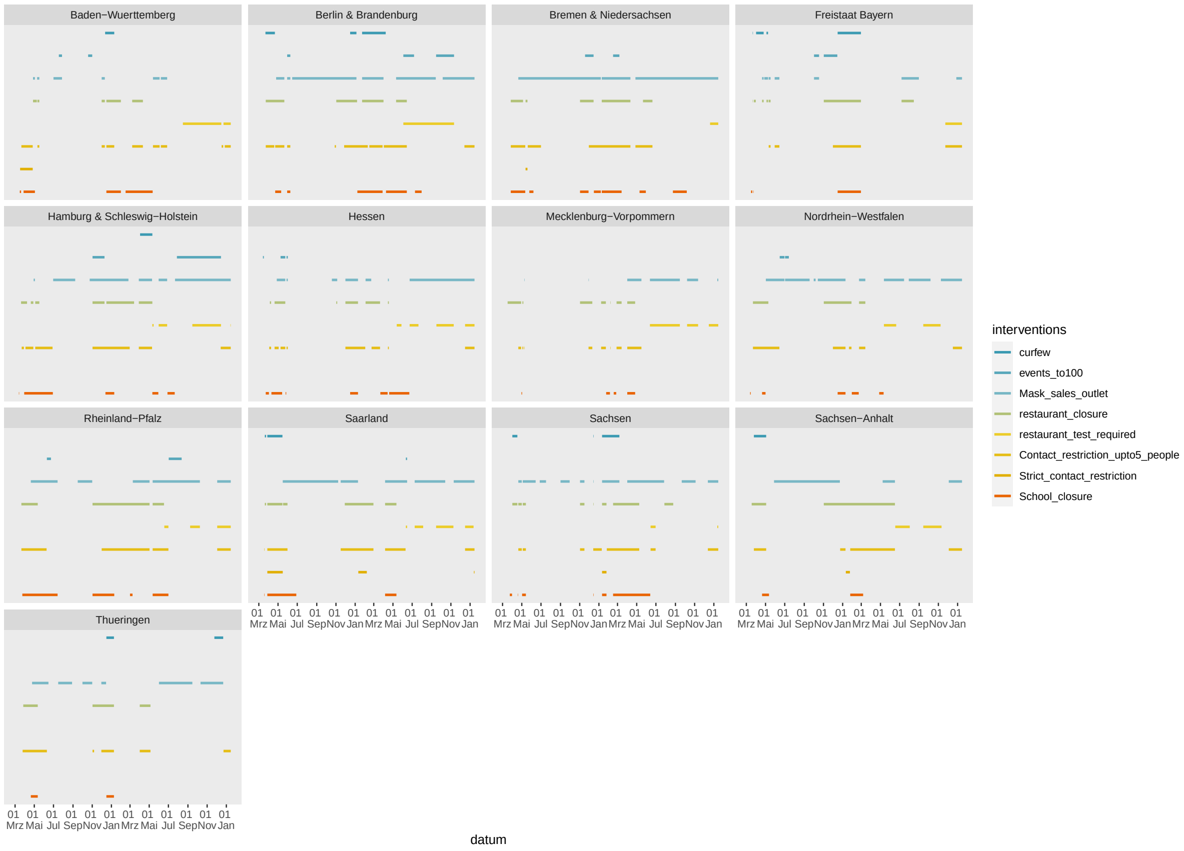

Figure S5: list of NPIs across 13 states when imputation was set to 0 and the subcategories were excluded

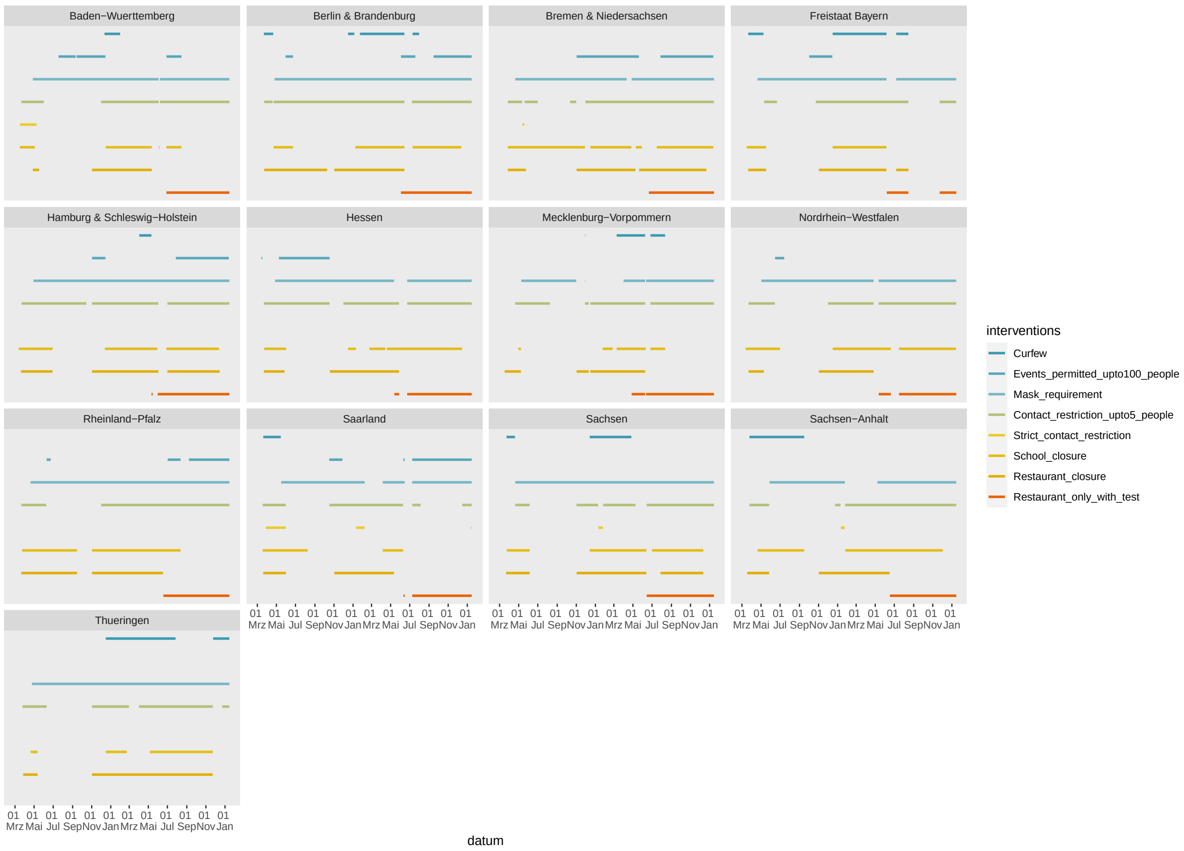

Figure S6: list of NPIs across 13 states when imputation was set to 1 and the subcategories were included

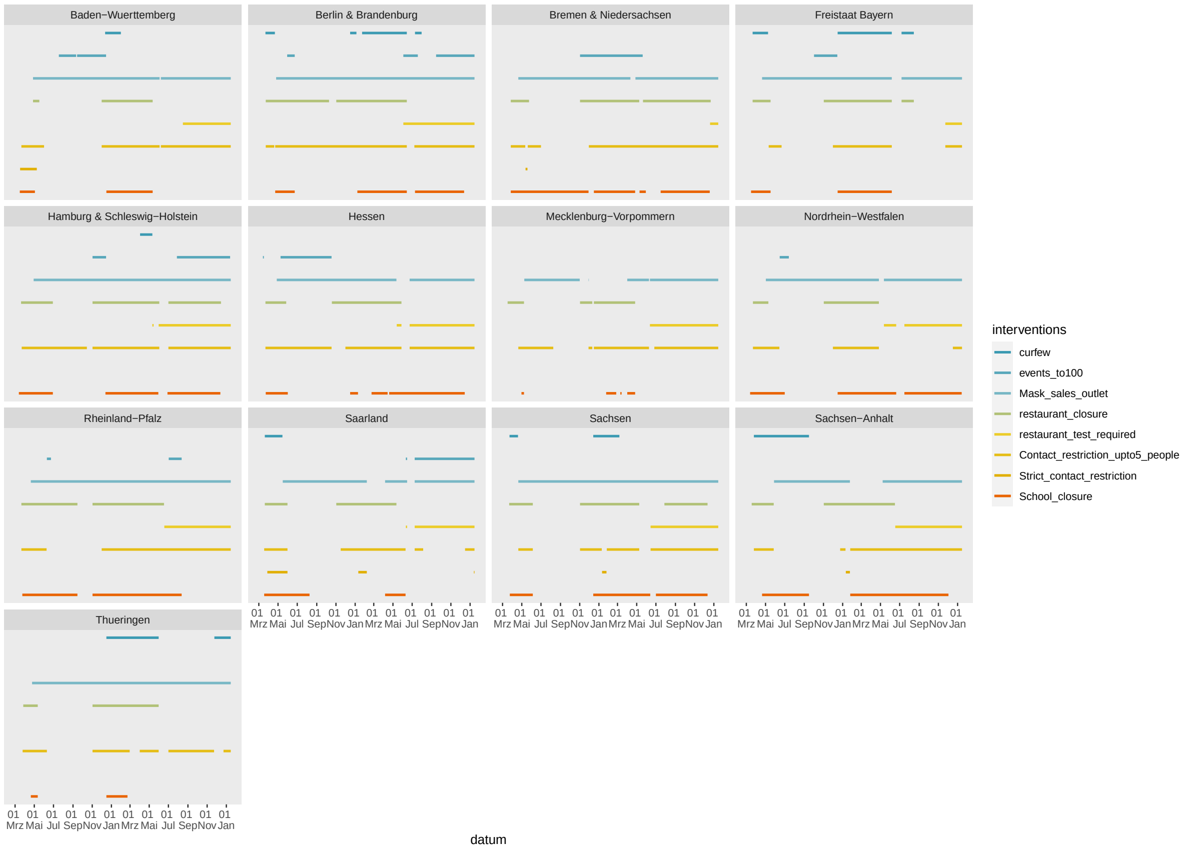

Figure S7: list of NPIs across 13 states when imputation was set to 1 and the subcategories were excluded

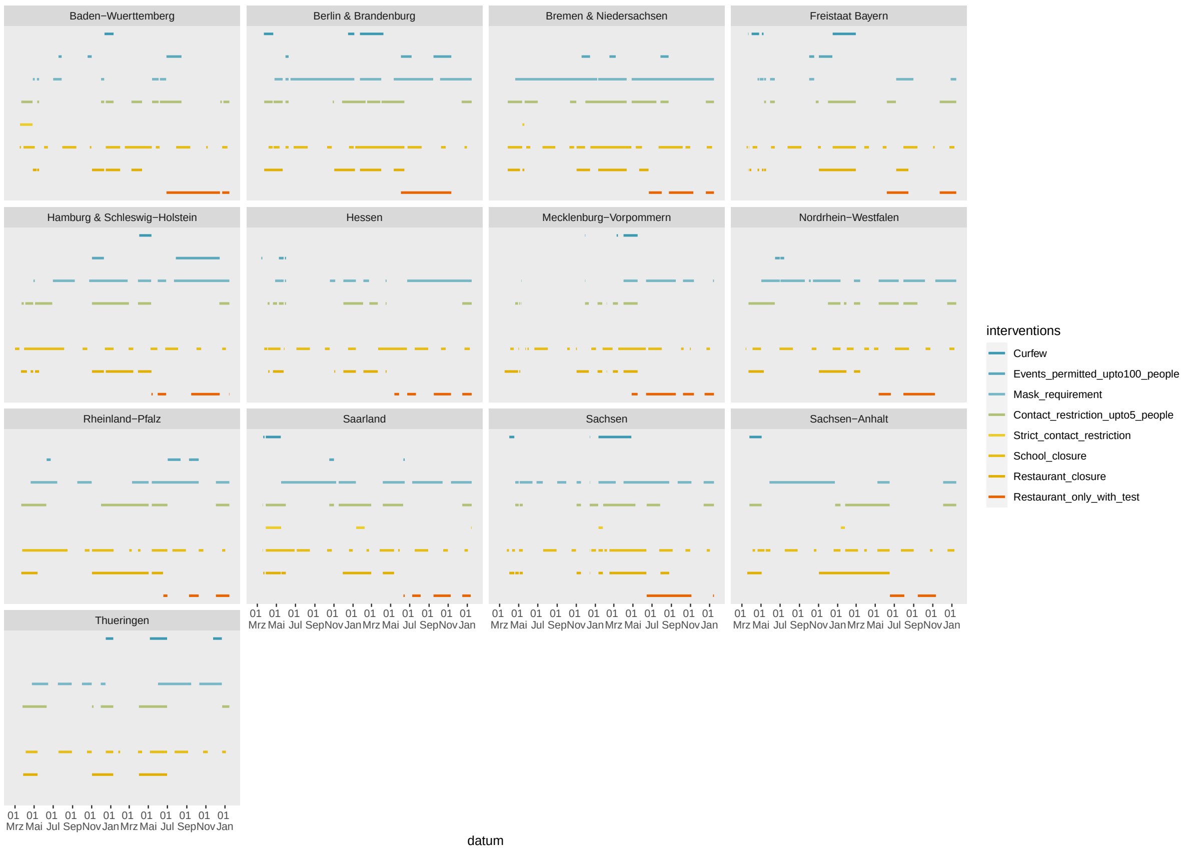

Figure S8: list of NPIs across 13 states when imputation was set to 0 and the subcategories were included

B Technical description of the model

In this section, we give a more technical description of the model. For a rigorous description with all details, we refer to Rehms et al. [15]. The model can be split into two parts: Inferring the unknown number of infections from known data and the estimation of the NPIs from these infections.

B.1 Inferring the infections

The number of infections is inferred by the following reported time series: deaths, cases, hospital admissions, and ICU occupations. This is done as follows. Firstly, the time from infection to symptom-onset is included (incubation time):

$$C_{t,m} = \sum_{u \leq t} I_{t,m} \left(F_{\xi^C}(t-u+1) - F_{\xi^C}(t-u) \right)$$

where $I_{t,m}$ is the number of infections as time t in federal state m (location), F_{ξ^C} is the incubation time distribution and $C_{t,m}$ is the resulting number of persons with symptoms. Note that this also includes persons with very light or even no symptoms. Given the cases, the model connects this quantity with the reported cases, deaths, and hospital data. This is done by four sub-models.

B.1.1 Reporting model

Given an actual case, this case does not get reported immediately, but with a delay. Moreover, testing capacities and strategies vary over time, which should also be reflected. This is done in the following way:

$$C^R_{t,m} \sim NegBinom\left(\mu^R_{t,m}, \phi^R\right)$$

where

$$\mu^R_{t,m} = \rho_{t,m} \sum_{u<t} C_{u,m} \left(F_{\xi^{R,w}_m}(t-u+1) - F_{\xi^{R,w}_m}(t-u) \right).$$

The reported number of cases for each time t follows a Negative Binomial distribution with an expected mean $\mu^R_{t,m}$ and a size parameter ϕ^R controlling the potential overdispersion. The expectation is given by a partial convolution of the past cases and time-shifting distributions $F_{\xi^{R,w}_m}$ which depends on weekday w and location m. This operation models the reporting delay with a seasonal pattern for specific weekdays (on Sundays, for instance, local authorities often report less or no data). $\rho_{t,m}$ represents a piece-wise constant reporting ratio that captures structural changes in the reporting due to varying testing.

B.1.2 Death model

Given an infected person with symptoms, this person dies from Covid-19 after some time with a certain probability. Mathematically this can be expressed as

$$D_{t,m} \sim NegBinom\left(\mu^D_{t,m}, \phi^D\right)$$

where

$$\mu^D_{t,m} = \pi^D_{t,m} \sum_{u \leq t} C_{u,m} \left(F_{\xi^{D,w}_m}(t-u+1) - F_{\xi^{D,w}_m}(t-u) \right)$$

where $D_{t,m}$ is the number of observed deaths, $F_{\xi^{D,w}_m}$ is the symptoms-to-deaths-distribution which reflects the time until a person with symptoms dies. $\pi^D_{t,m}$ is the infection fatality rate (IFR) which encodes the probability of dying given an infection. The IFR is a fixed parameter in the model and must be set with caution. Rehms et al. [15] incorporate the age structure, the effect of vaccinations, and the surge of more severe variants of concern which is also used for this application.

B.1.3 Hospitalization model

A case with symptoms may be hospitalized in the case of a more severe disease progression. As the deaths, this can be modeled in a similar way:

$$H_{t,m} \sim NegBinom\left(\mu^H_{t,m}, \phi^H\right)$$

where

$$\mu^H_{t,m} = \pi^H_{t,m} \sum_{u \leq t} C_{u,m} \left(F_{\xi^H}(t-u+1) - F_{\xi^H}(t-u) \right)$$

where F_{ξ^H} represents the distribution of time until a case gets hospitalized and $\pi^H_{t,m}$ is the probability. We use the same mechanics as in Rehms et al. [15], but include a new parameter β^H_{change} which is used to capture structural changes in the data from the 1st of July 2020. This is done by multiplying $\pi^H_{t,m}$ with β^H_{change} for all days after the 1st of July 2020. We estimate this parameter along with all other estimable parameters in the model to enable more flexibility and a better fit to the data.
The connection to ICU occupancy works in the same way, but with parameter $\pi^{ICU}_{t,m}$ and time shifting distribution $F_{\xi^{ICU}}$. As the ICUs are given as occupancy, this $F_{\xi^{ICU}}$ is a combination of the time until ICU admission and the actual ICU allocation.

B.2 Estimation of NPIs

Given the latent variable infections, one can derive the infection dynamics from that series. We model these dynamics using a renewal equation:

$$
\begin{aligned}
&I_{t,m} \sim NegBinom\left(\mu_{t,m}, \phi^I\right) \\
&\text{where} \\
&\mu_{t,m} = R_{t,m} \sum_{u<t} I_{u,m} \left(F_\gamma(t-u+1) - F_\gamma(t-u)\right).
\end{aligned}
$$

where the number of infections at location m at time t follows a Negative Binomial distribution with possible overdispersion. The expectation of this distribution is given as a function of past infections modeled via the generation time distribution in a partial convolution. This can be seen as a more flexible version of classical compartmental models The initial mean value of $I_{1,m}$ is given by tau_m where this expectation is modeled via a hierarchical model, i.e. $\tau_m \sim N^+(\tau, \sigma_\tau)$.
The reproduction number is explicitly given in the renewal equation. The NPIs are assumed to directly affect this quantity:

$$
R_{t,m} = R^0_{t,m} \exp\left(-\sum_{k=1}^{K+3} \alpha_{k,m} \cdot \mathbb{1}_{k,m}(t)\right) \cdot (1 - c^1_{t,m} - c^2_{t,m} \cdot (1 - c^1_{t,m}))
$$

with

$$
\begin{aligned}
c^1_{t,m} &= \frac{\sum_{u<t} I_{u,m}}{N_m} \cdot (1 - \beta^{reinf}) \\
c^2_{t,m} &= \frac{\sum_{u<t} (Vacc^1_{u,m} \cdot \beta^{vacc1} + Vacc^2_{u,m} \cdot \beta^{vacc2})}{N_m}.
\end{aligned}
$$

Here, $R_{t,m}$ is the reproduction which is a function of a basic reproduction number $R^0_{t,m}$, the effect of $K+3$ NPIs (three additional seasons with one as reference), and two correction factors. The basic reproduction number is assumed to be time-dependent as new variants of concern affect it. The correction factor $c^1_{t,m}$ reflects that already infected individuals can be reinfected only with some probability which is controlled by $beta^{reinf}$. The second correction factor $c^2_{t,m}$ controls for vaccination coverage in the population where two vaccinations are considered. Here, β^{vacc1} and β^{vacc2} represents the reduced transmissibility due to vaccinations and $Vacc^1_{u,m}$ and $Vacc^2_{u,m}$ is the number of infected individuals. These quantities are assumed to be the same as in Rehms et al. The effects of the NPIs are either active or not at each time t which is encoded by the indicator function $\mathbb{1}_{k,m}$. As well as the initial number of infections on day one, the NPIs are also modeled in a hierarchical manner:

$$
\alpha_{k,m} \sim N\left(\alpha_k, \sigma^2_{\alpha_k}\right).
$$

Hence, each federal state gets an individual estimate for each NPI effect, while sharing a common mean which can be interpreted as an overall effect. The basic reproduction number is modeled in the same way.

C Convergence diagnostics

This section shows the convergence diagnostics for the parameters of major interest. i.e. for the mean estimates of the NPIs α_k. Figure S9 shows the traceplots of the eight sampled Markov chains. Moreover, we show in table S1 the point estimate and the upper limit of the CI. Moreover, we show the posterior predictions compared to the used data in section E of this supplement.

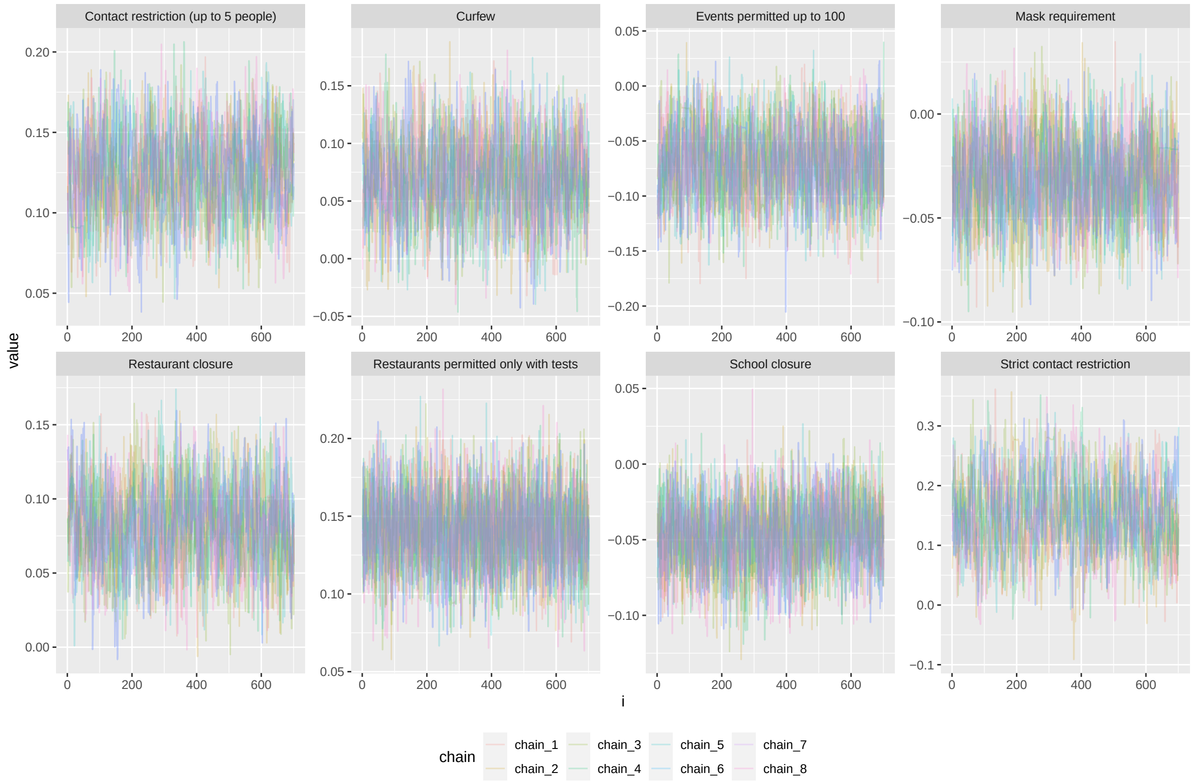

Figure S9: Trace plots for the α_k

Table S1: Table of the potential scale reduction factor (Point estimate and upper CI limit)

NPI	Point Estimate	Upper CI
Contact restriction (up to 5 people)	1.01	1.02
Strict contact restriction	1.01	1.03
Curfew	1.00	1.01
Events permitted up to 100	1.01	1.02
Mask requirement	1.01	1.02
Restaurant closure	1.01	1.03
Restaurants permitted only with tests	1.00	1.01
School closure	1.00	1.01

D Sensitivity analysis

We run sensitivity analysis to assess the impact of model assumptions:

- Allowing for "overreporting". We allow the value of the underreporting rate $\rho_{t,m}$ to be larger than one. A value above 1 can be interpreted as overreporting meaning that a test could be false positive. In a regime of many tests and low prevalence of the virus, this could be the case.
- A reduced value for the contagiousness of the variants of concern. We assume a high informative prior on the contagiousness of the variants of concern (see Rehms et al. [15].) As this may distort the estimation of the NPIs at specific time periods, we test the potential influence by reducing the mean of the prior.
- Modified infection fatality rate. As the IFR is a fixed quantity that links the series of deaths to the infections, we test whether multiplying this value with a scalar would change the results drastically.

Figures S10 (allowing for overreporting), S11 (reduced contagiousness) and S12 (modified IFR) present the estimated posterior for the three sensitivity analysis.

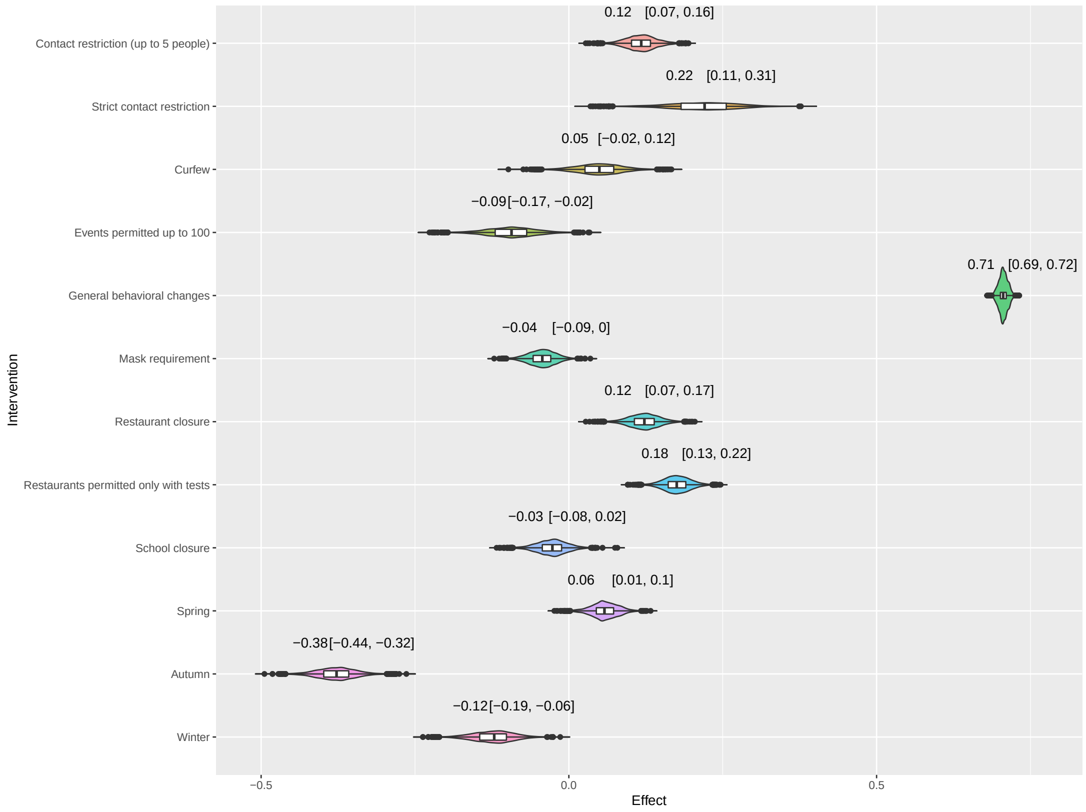

Figure S10: Estimated effects when allowing for overreporting.

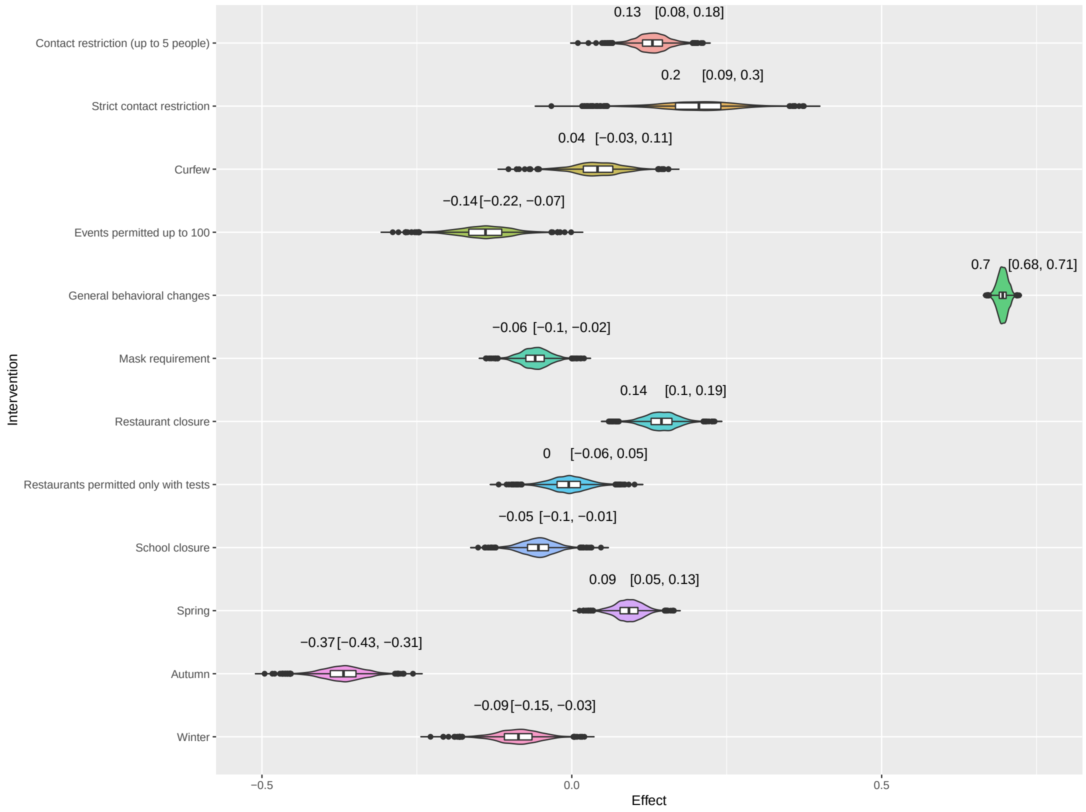

Figure S11: Estimated effects when the contagiousness of the variants of concern is reduced.

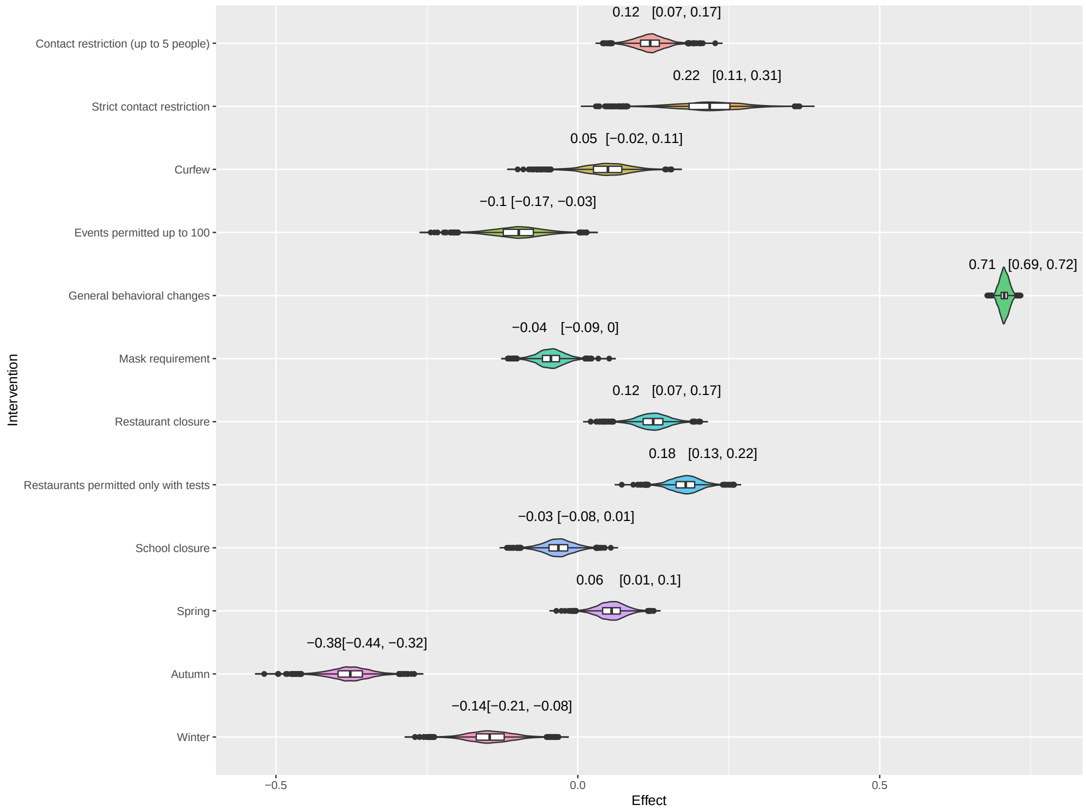

Figure S12: Estimated effects when the IFR is modified.

E Posterior predictive checks

In this section, we provide graphs of posterior predictions and the estimated infections for reported cases (Figure S13), deaths (Figure S14) hospitalizations (Figure S15) and intensive care unit occupancy (Figure S16) by state. Black encodes the observed time series, posterior mean, and 50%- and 95% -credible intervals are given in blue. The model recovers the underlying data quiet well for the majority of the time and states. However, for hospital and ICU data, the model suggests higher numbers (in particular in the second wave of the pandemic.)

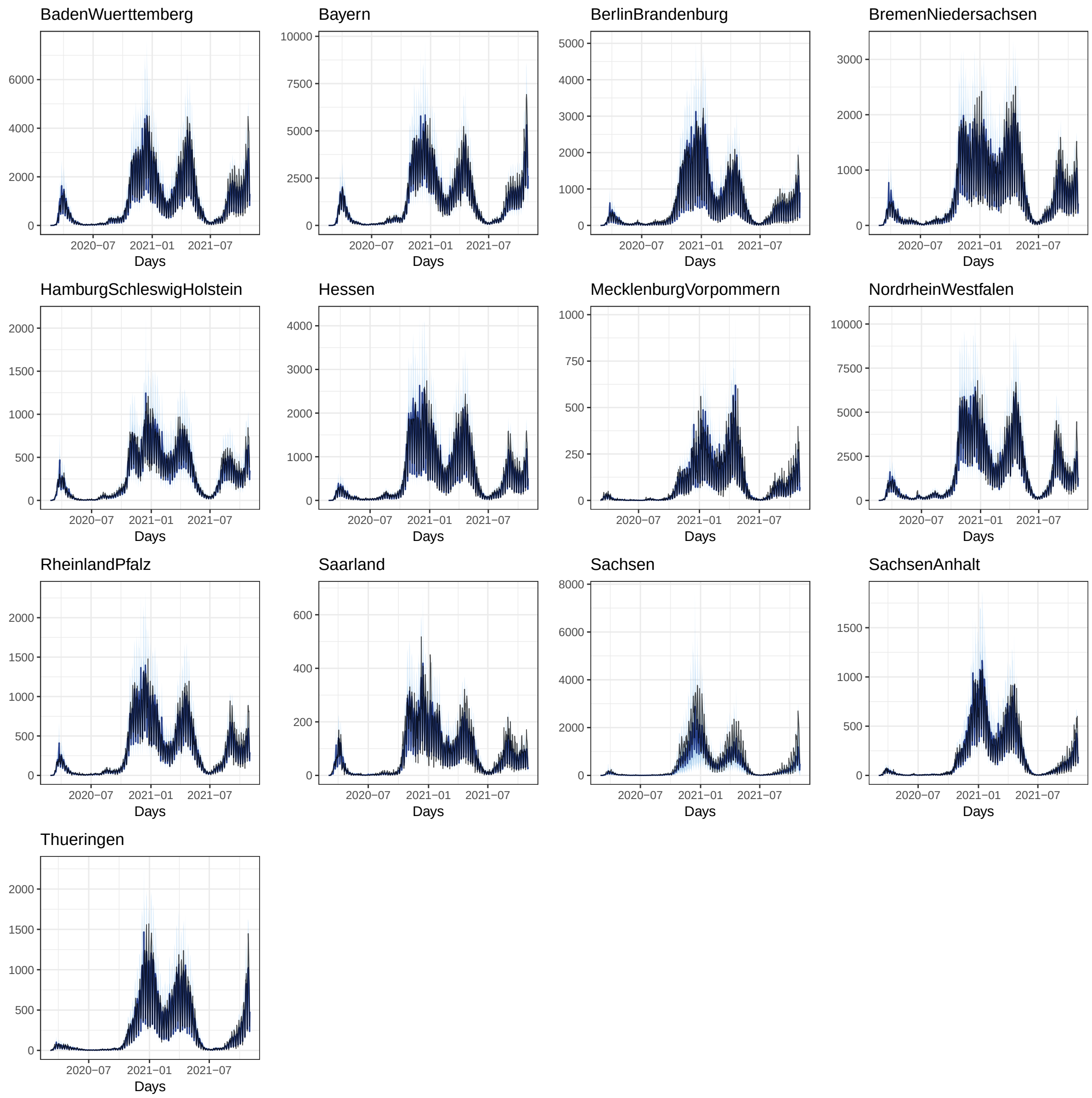

Figure S13: Posteriors predictions for reported cases

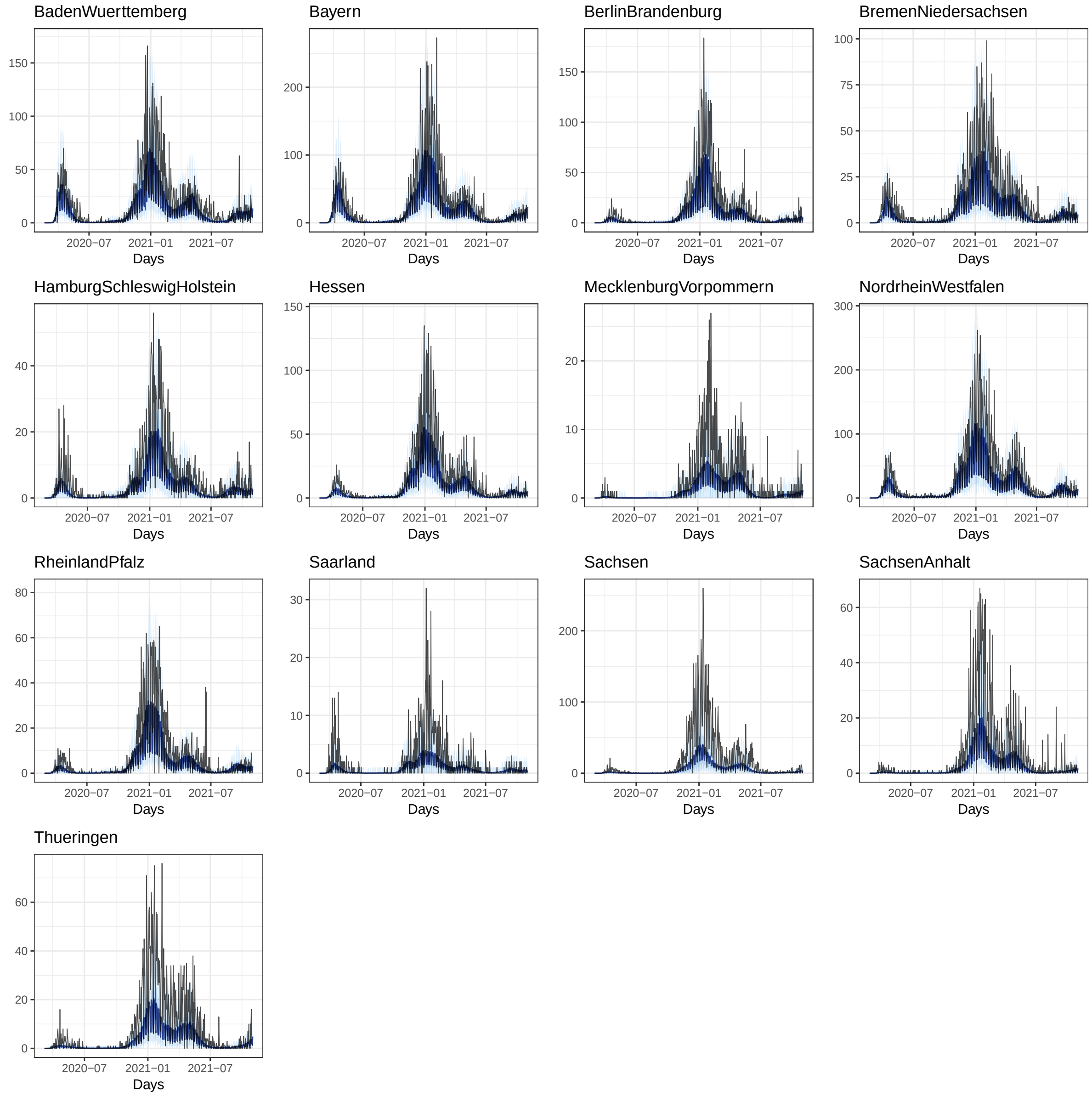

Figure S14: Posteriors predictions for reported deaths

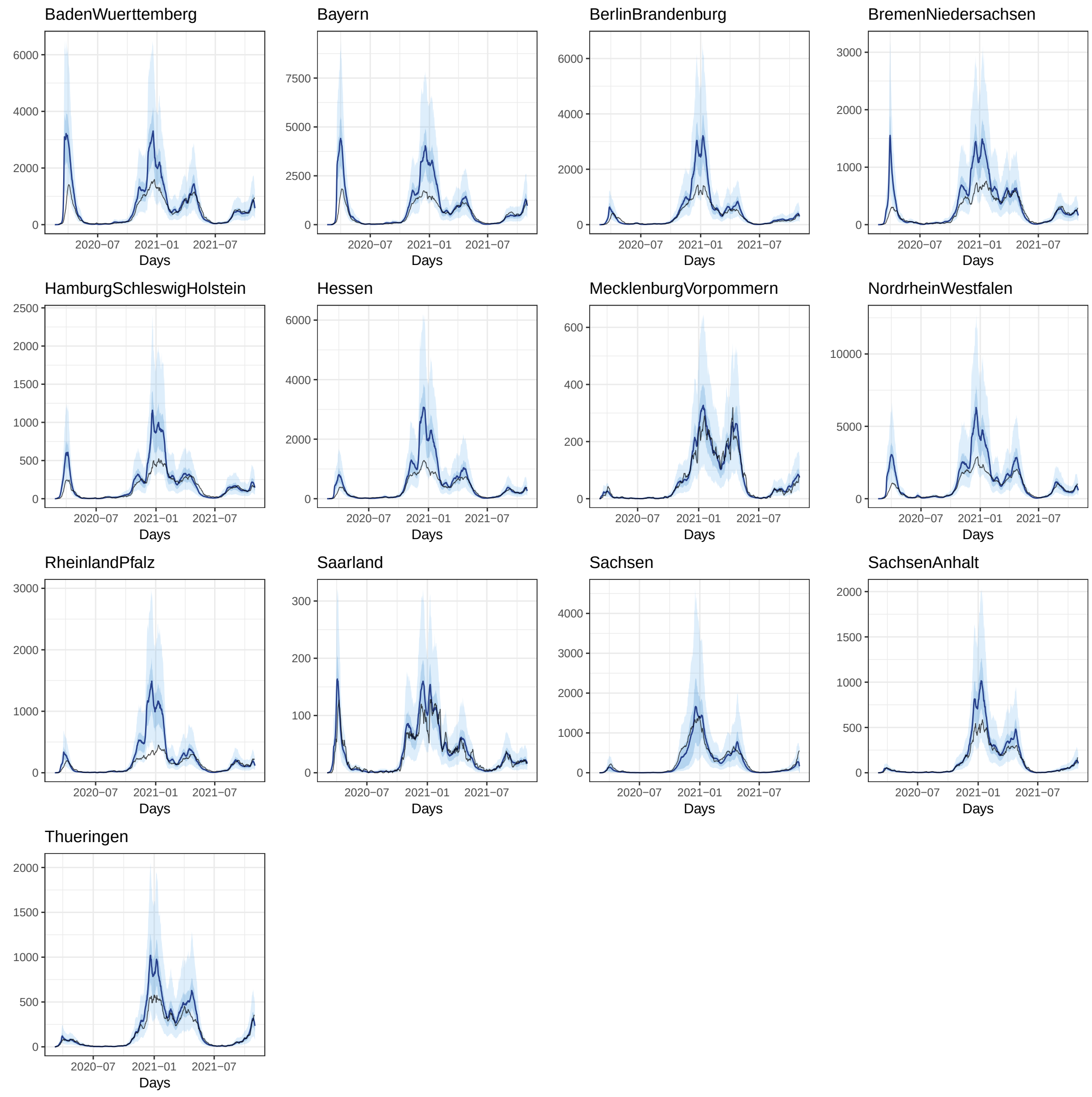

Figure S15: Posterior predictions for hospitalizations

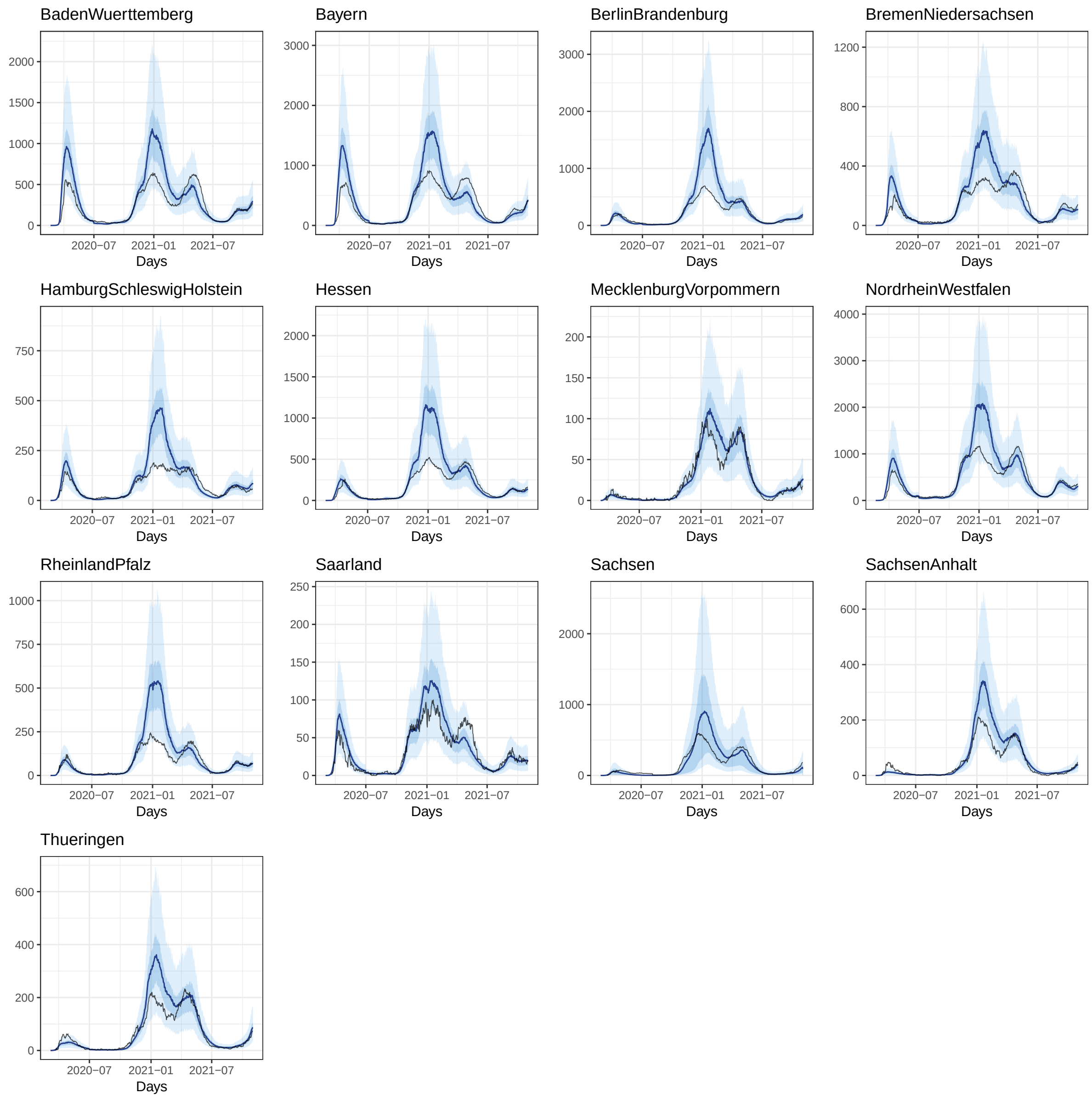

Figure S16: Posterior predictions for intensive care unit occupancy

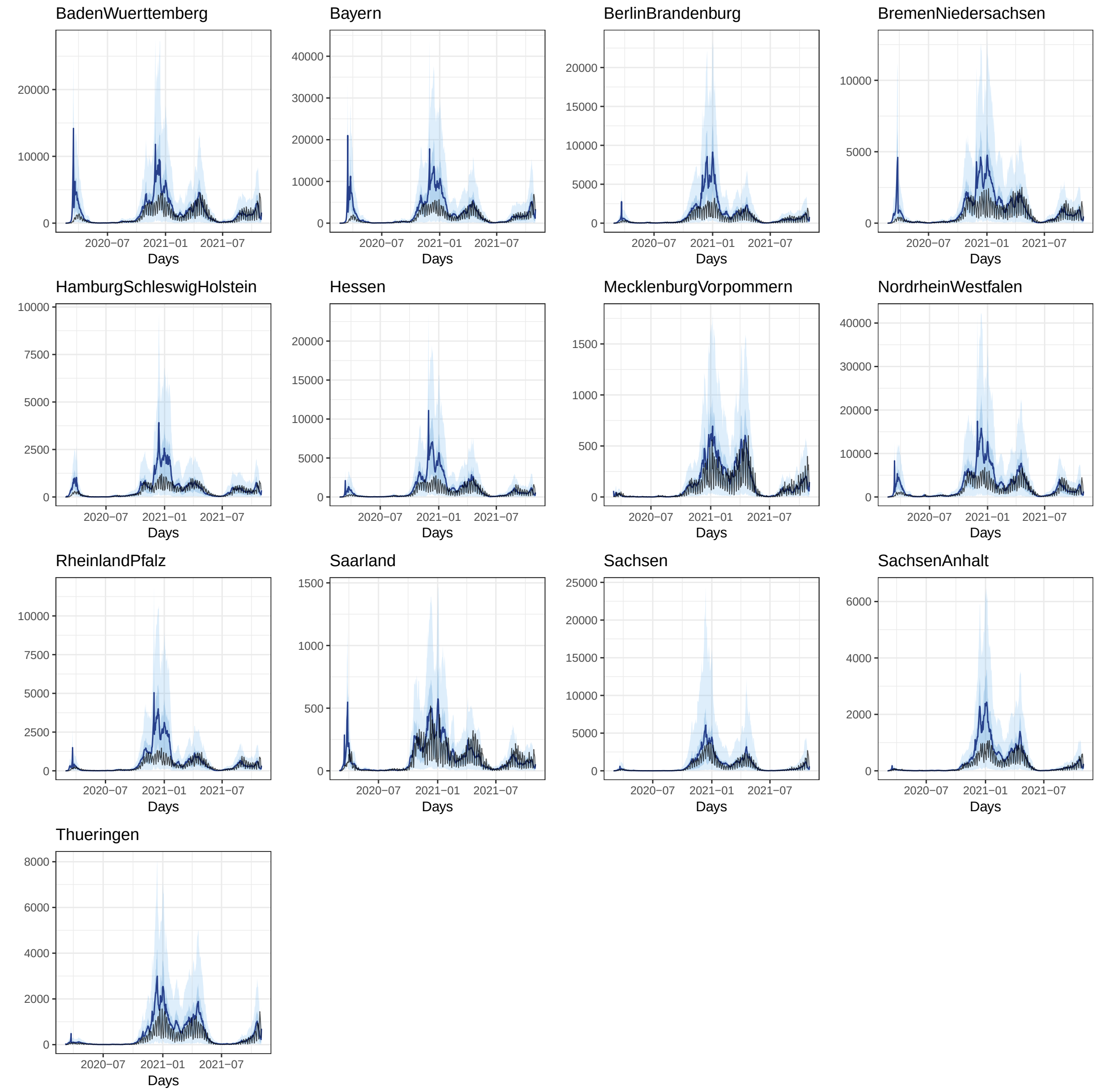

Figure S17: Estimated number of daily infections (in blue) and observed number of reported cases (in black)
